# Editorial: Nephrotic Syndrome in Pediatric Patients

**DOI:** 10.3389/fped.2017.00167

**Published:** 2017-08-03

**Authors:** Robert P. Woroniecki, Agnieszka Swiatecka-Urban, Frederick J. Kaskel

**Affiliations:** ^1^Stony Brook Children’s Hospital, Stony Brook, NY, United States; ^2^Department of Nephrology, Children’s Hospital of Pittsburgh, Pittsburgh, PA, United States; ^3^Department of Cell Biology, University of Pittsburgh School of Medicine, Pittsburgh, PA, United States; ^4^Division of Pediatric Nephrology, Children’s Hospital at Montefiore, Albert Einstein College of Medicine, New York, NY, United States

**Keywords:** podocytopathy, APOL1, FSGS, calcineurin inhibitor, outcomes, edema, endocytic trafficking, obesity

**Editorial on the Research Topic**

**Nephrotic Syndrome in Pediatric Patients**

Nephrotic syndrome (NS) remains a clinical diagnosis, encompassing proteinuria, dyslipidemia, hypoalbuminemia, and gravity-dependent edema. We came a long way since the initial gross descriptions of NS as dropsy, lipoid nephrosis, and nil disease to the most recent classification of podocytopathies as causes on NS. Recent advances demonstrate that mutations of a number of genes regulating structure and/or function of glomerular filtration barrier are causally associated with histologic diagnosis of focal segmental glomerulosclerosis (FSGS), diffuse mesangial sclerosis, and clinical unresponsiveness to glucocorticosteroids in patients with NS. These findings explain why a subset of children who do not respond to treatment have genetic causes of NS. While the genetic causes represent only a minority of childhood NS, the exact molecular mechanisms underpinning the steroid-responsive NS remain largely unknown. Newer data explain that remission of NS after treatment with glucocorticosteroids, and an overall favorable prognosis are not consistent across all pediatric age groups and ethnic backgrounds.

In this special pediatric nephrology series, we have combined contributions from experts in childhood NS working in a variety of fields, including basic science, clinical medicine, and epidemiology to provide the reader with a comprehensive and the most up-to-date information on different aspects of NS. We were fortunate to enlist a prominent group of 31 authors to contribute a wide range of articles. In total, 10 papers have been included, with 3 original research articles, 6 reviews, and 1 historical perspective (Pal and Kaskel; Ranganathan; Swiatecka-Urban; Ellis; Hjorten et al.; Beins and Dell; Spino et al.; Chanchlani and Parekh; Woroniecki et al.; Canetta and Radhakrishnan).

We introduced the topic by historical perspective authored by Pal and Kaskel. The article walks readers through inspiring stories of tremendous efforts and international collaborations pawing the milestones in understanding of the pathophysiology and treatment of NS. Progress in the field is measured by a huge decrease in mortality related to NS from above 50% to less than 3% in the span of half of a century. As authors note, continued multicenter collaborations are critical to achieve new milestones and progress to personalized approach in the treatment of this rare childhood disease. Ranganathan reviewed the histopathology of congenital NS, minimal change disease, and its variants, and FSGS (Ranganathan). Swiatecka-Urban focused on the role of endocytic trafficking at the mature podocyte slit diaphragm and reviewed the critical role of signaling in maintaining the integrity of slit diaphragm that protects from to glomerular protein loss (proteinuria), manifesting as NS. Ellis described current pathophysiology of edema formation in NS (Ellis). The author reviewed “the under and overfill hypothesis” and current evidence for loss of plasmin and other serine proteases in the urine resulting in upregulation of the epithelial sodium channel (ENaC) causing Na+ and fluid retention (Ellis). Hjorten et al. reviewed the long-term outcomes of NS in childhood noting the importance of steroid responsiveness as a prognostic indicator. While steroid-resistant NS (SRNS) is associated with a high-risk (HR) of developing end-stage renal disease (ESRD), long-term complications of steroid-sensitive NS are under-recognized (Hjorten et al.). Beins and Dell in their retrospective chart review surmise that although majority of SRNS patients initially respond to calcineurin inhibitor therapy, a significant percentage still progresses to ESRD, despite achieving short-term remission. Spino et al. reviewed current therapeutic options for FSGS and discussed the problem of varied efficacy related to specific therapy, patient characteristics, cost, and common side effects, and noted that this variability of response to treatment is likely caused, at least in part, by the heterogeneity in the etiology of FSGS.

Chanchlani and Parekh further reviewed the emerging role of genetic factors determining the response to treatment within an ethnic group, noting that among African-Americans (AA), the risk variants in *APOL1* are associated with a more than 10-fold increase in risk of FSGS and HR carriers have a twofold greater risk of progression to ESRD. Woroniecki et al. presented original data indicating that AA children with HR *APOL1* genotype and FSGS have increased prevalence of obesity and left ventricular hypertrophy (LVH) despite a later age of FSGS onset, while adjusting for socioeconomic status. They noted that treatment of obesity may be an important component of chronic kidney disease and LVH management in this population (Woroniecki et al.). Finally to contrast with children, Canetta and Radhakrishnan reviewed the evidence-based approach to adult-onset NS.

Our overarching goal was to stimulate interest of academic and practicing physicians and scientists in this relatively rare childhood disease with diverse pathophysiology and outcomes. We hope that we will be seeing more studies and research in this field in the near future.

## Author Contributions

RW wrote and reviewed the editorial, AS-U and FK contributed to review process and editing.

## Conflict of Interest Statement

The authors declare that the research was conducted in the absence of any commercial or financial relationships that could be construed as a potential conflict of interest. The handling editor declared a shared affiliation, though no other collaboration, with one of the authors AS-U and states that the process nevertheless met the standards of a fair and objective review.

